# Thermophilic bacteria of the Arabian Gulf and their emerging biotechnological applications: current insights and future prospects

**DOI:** 10.3389/fmicb.2026.1865082

**Published:** 2026-08-20

**Authors:** Amjad Khalil, Sa-ad Abubakari

**Affiliations:** Department of Bioengineering, King Fahd University of Petroleum and Minerals, Dhahran, Saudi Arabia

**Keywords:** Arabian Gulf, biotechnological applications, extremophiles, thermophilic bacteria, thermostable enzymes

## Abstract

Thermophilic bacteria represent a powerful class of extremophiles whose ability to thrive at elevated temperatures makes them indispensable to modern biotechnology. The Arabian Gulf characterized by extreme heat, geothermal systems, hot springs, oil reservoirs, and hypersaline habitats hosts a rich yet understudied reservoir of these organisms. This review consolidates current insights into the diversity, ecological niches, and biotechnological relevance of thermophilic bacteria isolated from the region. Dominant genera such as *Bacillus*, *Geobacillus*, *Thermus*, *Anoxybacillus*, and *Brevibacillus* exhibit remarkable physiological and molecular strategies that enable survival under intense thermal, saline, and pH stress. Their capacity to produce thermostable enzymes, including proteases, amylases, lipases, cellulases, and DNA polymerases, positions them as high-value contributors to sectors spanning bioenergy, pharmaceuticals, food processing, agriculture, and environmental remediation. Beyond enzyme production, emerging applications such as antimicrobial compound discovery, hydrocarbon bioremediation, wastewater treatment, and sustainable bioprocessing highlight the region’s untapped biotechnological potential. However, systematic exploration remains limited, hindered by sparse isolation efforts, incomplete physiological profiling, and a lack of genomic and omics-driven studies. The review underscores the need for integrated approaches that merge classical microbiology with advanced molecular and systems-level tools. Collectively, thermophilic bacteria from the Arabian Gulf constitute a promising yet underutilized biological resource poised to drive sustainable industrial innovation and environmental solutions.

## 1 Introduction

Thermophilic bacteria represent a unique class of microorganisms capable of thriving at elevated temperatures exceeding 70 °C, with remarkable resilience and adaptability that sets them apart from mesophilic organisms (Kumari et al., 2024). These heat-loving microorganisms exhibit high metabolic activity and possess unique adaptations that enable survival in extreme thermal conditions found in hot springs, geothermal fields, and deep-sea hydrothermal vents (Pandey and Dhakar, 2025). The exceptional ability of thermophiles to survive in such inhospitable environments has been attributed to their specialized cellular and molecular mechanisms, including stabilized membrane structures, thermostable proteins, and sophisticated DNA repair systems. They inhabit diverse locations such as hot springs, deep-sea hydrothermal vents, and geothermal soils (Kochetkova et al., 2022). Indonesia, located along the Pacific Ring of Fire, provides an ideal case study for thermophilic bacterial diversity, with abundant geothermal sites and hot springs hosting diverse thermophilic communities (Wulandari et al., 2025). Similarly, extreme thermal environments both natural (hot springs, fumaroles, geysers) and man-made (compost heaps, coal refuse piles) serve as rich reservoirs of these thermophilic microorganisms, making them valuable sources of thermostable and thermoactive enzymes (Pandey and Dhakar, 2025).

The geographical distribution of thermophilic bacteria spans diverse regions globally, with particularly high concentrations in geothermal zones. Studies on the Indian Himalayan Region documented thermophilic bacteria growth ranging between 40 and 100 °C, with major bacterial species including *Bacillus* spp., *Geobacillus* spp., *Paenibacillus* spp., and *Pseudomonas* spp. (Verma et al., 2025). The northern region of Chile has shown unique polyextreme habitats supporting diverse thermophilic communities in the Atacama Desert and Andean high plains, where isolates demonstrated significant hydrolytic enzyme activities (Valenzuela et al., 2024). The Arabian Gulf region, while understudied compared to other geothermal zones, presents unique environmental conditions that could harbor novel thermophilic bacterial species. The extreme temperature gradients, high salinity, and oligotrophic conditions characteristic of this region create distinctive selective pressures for microbial adaptation (Al-Thani and Yasseen, 2021; Lachkar et al., 2025).

Thermophiles play crucial roles in biogeochemical processes like organic matter decomposition and methane production. Moreover, their unique enzymes hold promise for various biotechnological applications, such as biofuel production and drug discovery. Despite the harsh conditions they endure, thermophiles offer valuable insights into the boundaries of life on Earth and the potential for life in analogous extreme environments on other planets (Carré et al., 2022).

Thermophiles, such as *Geobacillus*, *Alicyclobacillus*, and *Anoxybacillus*, produce enzymes that present an alternative solution to various challenges encountered in industrial biocatalysis. Referred to as thermozymes, these enzymes exhibit robustness, combined with both thermostability and thermoactivity, enabling them to function efficiently in high-temperature bioprocesses (Regassa et al., 2021).

They also demonstrate resilience to elevated pressure and solvents that typically denature proteins, common conditions in industrial settings. These distinctive traits set them apart from their mesophilic counterparts (Aggarwal et al., 2024). Additionally, utilizing higher temperatures in industrial operations enhances solubility of substrates and products, reduces hydrolysis duration, and lowers the risk of microbial contamination. Consequently, thermostable enzymes are highly sought-after as biocatalysts (Arya et al., 2021). Furthermore, enzymes from hyperthermophiles, usually archaea capable of thriving at temperatures exceeding 80 °C, exhibit structure-function relationships that confer exceptional thermostability and peak activity at temperatures surpassing 70 °C, with certain enzymes even retaining catalytic efficacy above 100 °C.

The Arabian Gulf region represents one of Earth’s most extreme and unique aquatic environments, characterized by elevated temperatures, high salinity, intense solar radiation, and complex hydrocarbon-rich sediments. These harsh environmental conditions have shaped a specialized microbial ecosystem dominated by thermophilic and halophilic microorganisms adapted to thrive where few other organisms can survive. Therefore, this review examines the current status and future prospects of thermophilic bacteria in the Arabian Gulf, with emphasis on their diversity, adaptation mechanisms, and significant biotechnological potential for industrial applications.

A comparison of the key characteristics of the thermophilic *Actinomycetes*, *Bacillus*, and *Geobacillus* genera is shown in Table 1.

**TABLE 1:** Comparison of key characteristics among *Actinomycetes*, *Bacillus*, and *Geobacillus* Genera.

Feature	*Actinomycetes*	*Bacillus*	*Geobacillus*
Temperature range	45–75 °C	Up to 60 °C	45–75 °C
Applications	Soil antibiotics, Antifungals	Biotechnology, Enzyme production	Prospective anti-tumor agents, Food-safe AMPs
Useful compounds from some of the thermophiles	Peptaibiotics, Polyenes	Lipopeptides, Bacteriocins	Geobacillin types

The table compares selected thermophilic bacterial genera *Actinomycetes*, *Bacillus*, and *Geobacillus* highlighting their temperature ranges, major biotechnological applications, and bioactive compounds. *Actinomycetes* are renowned for producing antibiotics and antifungal metabolites, *Bacillus* species for synthesizing industrial enzymes, lipopeptides, and bacteriocins, and *Geobacillus* for generating thermostable antimicrobial peptides and potential antitumor compounds. This comparison underscores the significant industrial, pharmaceutical, and biotechnological value of thermophilic bacteria as sustainable sources of novel bioactive molecules and thermostable biocatalysts (Alhanbali et al., 2025).

## 2 Review methodology and literature selection

Literature searches were undertaken across PubMed, Scopus, Web of Science Core Collection, and Google Scholar from database inception. Search strategies combined taxonomic and functional descriptors “*thermophile*,” “thermotolerant,” “extremozyme,” “*hot spring*” with geographic terms encompassing the Arabian Peninsula and Gulf region “*Arabian Gulf,” “Persian Gulf,” “Saudi Arabia,” Kuwait, Qatar, Bahrain, Oman, “United Arab Emirates,*” GCC as well as terminology related to genomics, metagenomics, enzymology, hydrocarbon degradation, and bioremediation.

Peer-reviewed primary studies were eligible for inclusion when they reported microorganisms, genomes, microbial communities, enzymes, or biotechnological applications derived from samples collected within the Arabian Gulf or the broader GCC region. Selected review articles and nonregional primary studies were incorporated solely to provide clearly delineated comparative or mechanistic context and were not treated as evidence for local taxa or processes.

Studies were excluded when sampling locations were ambiguous, when the subject matter did not pertain to environmental thermophiles, when sources lacked peer review, or when nonregional findings were presented as direct support for Arabian Gulf systems. Titles, abstracts, and full texts were screened independently, with discrepancies resolved through consensus. As this work constitutes a narrative review, no formal meta-analysis or quantitative synthesis was performed.

In this review, the term “Arabian Gulf evidence” denotes research conducted on biological materials originating from the Arabian Gulf marine system, including its coastal zones and associated subsurface environments. By contrast, geothermal springs, desert habitats, and other non-marine sites within GCC countries are treated under a distinct category of “adjacent regional evidence.” Studies generated outside both the Arabian Gulf and the broader GCC region are incorporated solely to elucidate underlying mechanisms or to provide comparative context; they are not considered demonstrative of the presence, ecological roles, or functional capacities of local taxa.

The designation “thermophile” is applied exclusively to microorganisms exhibiting an optimum growth temperature above approximately 45 °C, whereas taxa capable of surviving elevated temperatures but displaying maximal growth below this threshold are classified as thermotolerant. Hyperthermophiles, typically characterized by growth optima exceeding 80 °C, are addressed separately when relevant to the thematic scope of this review.

With regard to table generation (1, 2, 3, 4, 5, and 6), studies were included if sampling occurred within the stated region and if the article reported temperature/growth, taxonomy, enzyme activity, or application data relevant to the table. Data were compiled from the cited primary studies, and values were not pooled due to differences in methods and conditions where appropriate.

## 3 Environmental characteristics and ecological context of the Arabian Gulf

### 3.1 Temperature regimes and physicochemical conditions

The Arabian Gulf, also known as the Persian Gulf in some international contexts, is one of the world’s most challenging marine environments. The region is characterized by extreme temperature fluctuations, high salinity, and significant anthropogenic pressures. The region experiences surface water temperatures that regularly exceed 35–40 °C during summer months, with some areas reaching even higher temperatures due to shallow depths and limited water exchange. The Gulf’s unique hydrographic characteristics, combined with evaporative processes and minimal freshwater inputs, create hypersaline conditions that range from approximately 37 to over 40 ppt, significantly exceeding typical oceanic salinity (Paparella et al., 2022).

Long-term environmental monitoring in the Arabian Gulf indicates persistent thermal stress on marine ecosystems, with projections suggesting further temperature increases as a consequence of climate change (Hereher, 2020; López et al., 2024). These conditions create a selective environment where only specially adapted microorganisms can thrive, driving the evolution of thermophilic and halophilic-thermophilic bacterial communities. The combination of temperature stress, elevated salinity, and limited nutrient availability establishes ecological niches that promote the development of robust extremophilic microbial populations. For instance, Sabkhas (salt flats) in NEOM, Saudi Arabia, are ecologically important yet fragile arid ecosystems that contribute significantly to biodiversity conservation and overall ecosystem functioning. However, increasing urbanization and anthropogenic activities pose serious threats to these environments, potentially altering their soil microbial communities. Microeukaryotes, in particular, are key indicators of ecosystem health and resilience, yet their diversity in Sabkhas remains poorly understood due to limited molecular-based studies (Gopi et al., 2025).

Compared with Mediterranean geothermal systems, where thermophilic communities are often structured around localized hydrothermal venting and steep geochemical gradients, the Arabian Gulf presents a broader polyextreme setting shaped by shallow-water heating, high evaporation, hypersalinity, hydrocarbon-rich sediments, and strong anthropogenic pressure from oil and gas activities (Somoza et al., 2021). The Red Sea also contains warm and saline marine environments, including brine pools and hydrothermally influenced sites; however, the Arabian Gulf is distinguished by its extremely shallow semi-enclosed basin, restricted water exchange, recurrent summer temperatures exceeding typical oceanic values, and close association with coastal Sabkhas and petroleum reservoirs (Rasul et al., 2015). These combined characteristics make the Arabian Gulf a distinctive natural laboratory for studying thermophilic and thermo-halophilic microorganisms with industrially relevant stress tolerance.

### 3.2 Anthropogenic stressors and their role in microbial ecology

The Arabian Gulf faces chronic pollution from oil and gas extraction, refining, and shipping activities, with cumulative impacts on microbial communities throughout the water column and sediments (Al-Mutairi, 2025). Heavy metal contamination, thermal pollution from desalination facilities, and hypersaline effluent discharge create additional stressors that fundamentally alter microbial community composition and function. These multiple stressors have collectively shaped the evolutionary trajectory of thermophilic bacteria in the Gulf, selecting for organisms capable of simultaneously tolerating elevated temperatures, high salinity, and hydrocarbon exposure.

## 4 Habitat characterization of thermophiles across the GCC region

Organisms known as thermophiles are those that can tolerate temperatures exceeding 45 °C. Thermophiles may be found in a variety of environments in the Gulf Cooperation Council (GCC), which is made up of Bahrain, Kuwait, Oman, Qatar, Saudi Arabia, and the United Arab Emirates (Zhu et al., 2021).

Due to the harsh environmental factors in the area, such as high temperatures, little rainfall, and high salt, these extremophiles have evolved to survive. An overview of the thermophile habitats in the GCC regions, such as hot springs, geothermal zones, and oil reserves, is given in the discussion that follows (Song et al., 2022).

### 4.1 Hot springs

In the GCC, hot springs are one of the thermophiles’ most well-known habitats. Hot water that frequently has a significant mineral concentration is present in these geothermal phenomena. The GCC has hot springs that may reach temperatures of above 50 °C, which is perfect for thermophilic bacteria. Scientists and researchers who study extremophiles and their unique adaptations are very interested in the existence of thermophiles in these hot springs (Parihar and Bagaria, 2019).

A study revealed a variety of thermophilic microbe communities may be found in the hot springs of the Gulf Cooperation Council (GCC), including those in Oman and Saudi Arabia (Aljohani, 2019). Numerous unique thermophilic bacterial strains that are suited to the high temperatures and particular geochemical circumstances of these hot springs were found to be present in the research (Massello et al., 2020).

### 4.2 Geothermal areas

For thermophiles in the GCC, geothermal sites are key habitats in addition to hot springs. High-temperature subsurface habitats, frequently linked to volcanic activity, are what define these regions (Minissale et al., 2019). Countries such as Oman and Saudi Arabia have geothermal resources that offer special environments for thermophilic bacteria to flourish. These geothermal regions’ high temperatures and unique chemical compositions favor creatures with particular heat- and chemical-tolerant adaptations (Noell et al., 2022 These settings serve as natural laboratories for studying the adaptability and biotechnological potential of thermophilic bacteria (Peng et al., 2024; Gupta et al., 2025).

### 4.3 Oil reservoirs

In the GCC, oil reservoirs provide important ecological niches for thermophilic bacteria. The extreme temperatures and pressures seen in these underground habitats are favorable for thermophilic bacteria. The petroleum industry is affected by the presence of thermophiles in oil reservoirs because these organisms can affect reservoir souring and oil quality. It is important to comprehend the populations of thermophilic microbes in oil reservoirs in order to manage reservoirs effectively and maximize oil recovery (Okoro et al., 2023; Pannekens et al., 2019).

**FIGURE 1:**
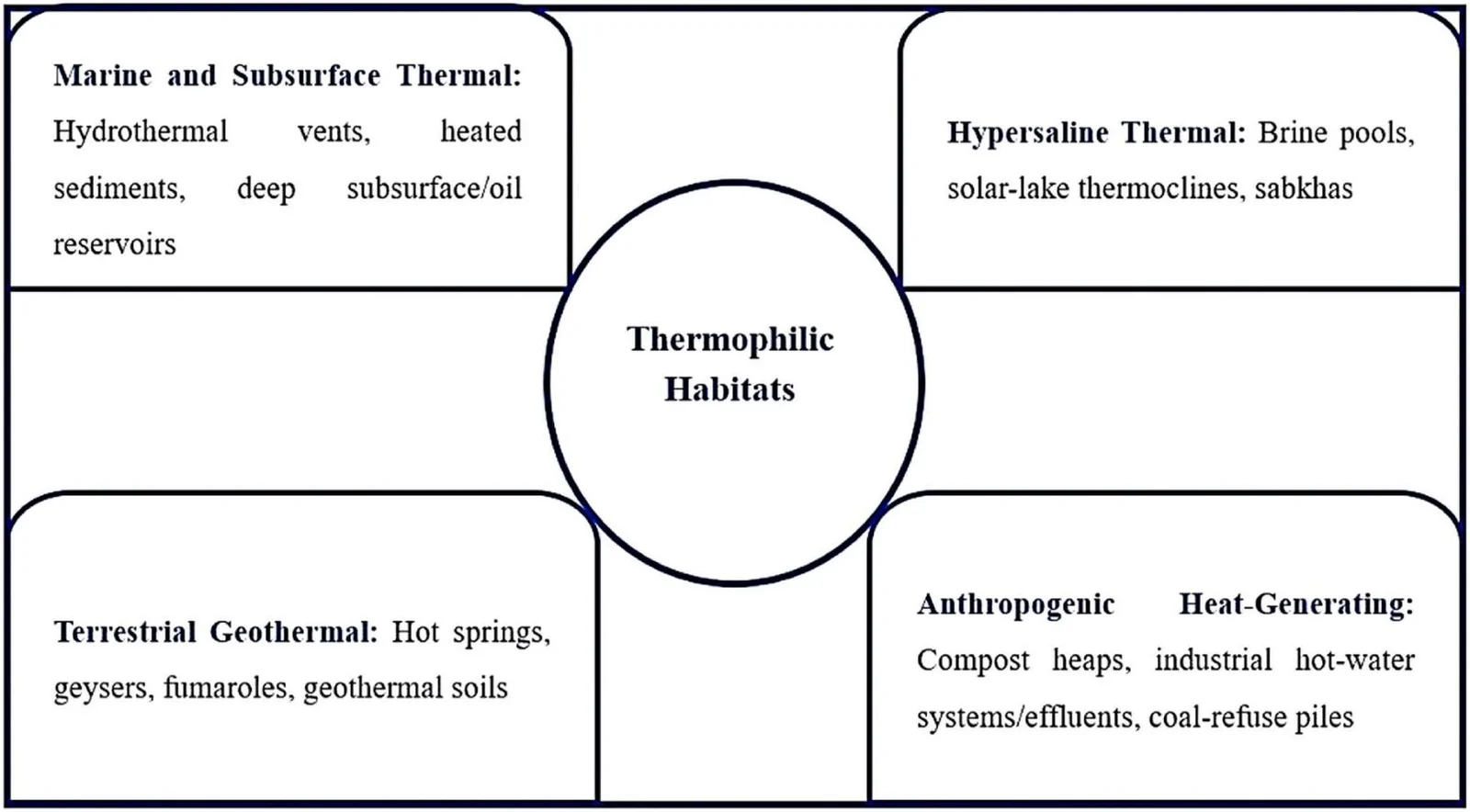
Representative habitats of thermophilic and thermotolerant microorganisms. The figure represents natural and anthropogenic habitats occupied by thermophilic and thermotolerant microorganisms. The categories are illustrative, not quantitative, and the size and position of the symbols do not indicate habitat area, abundance, or ecological importance. The habitats shown include terrestrial geothermal systems, marine and subsurface thermal systems, hypersaline thermal systems, and anthropogenic environments that generate heat.

In the GCC, thermophiles live in a range of harsh settings, including oil reservoirs, geothermal zones, and hot springs. These creatures have developed special adaptations to survive in harsh chemical environments and high temperatures. Researching the habitats of thermophiles in the Gulf Cooperation Council (GCC) has promise for biotechnological applications in a range of sectors, in addition to furthering our knowledge of extremophile ecology (Alrumman et al., 2018). The thermophilic bacteria habitats, both natural and anthropogenic, are presented in Figure 1.

## 5 Unraveling the diversity, taxonomic structure, and regional distribution of thermophilic bacteria across environmental gradients in GCC countries

### 5.1 Major bacterial lineages and phylogenetic groups

Thermophilic bacteria isolated from Arabian Gulf sediments and water samples predominantly belong to the genera *Geobacillus, Bacillus, Parageobacillus*, and *Anoxybacillus* (Adlan et al., 2020). These spore-forming bacteria represent the most abundant culturable thermophilic fraction in the region, reflecting their superior survival capabilities under extreme conditions. The discovery of thermophilic bacterial strains isolated from seawater and oil-contaminated soil samples within the Arabian Gulf, including species such as *Geobacillus kaustophilus*, *Bacillus jurassicus*, and *Parageobacillus caldoxylosilyticus*, has expanded our understanding of the thermal ecology of the region.

Metagenomic investigations of Arabian Gulf sediments and marine environments have revealed the presence of novel thermophilic taxa previously unknown or rarely encountered in this geographic region. These studies employ 16S rRNA gene sequencing and comparative genomics to classify isolates and assess their evolutionary relationships to known thermophilic bacterial lineages (Matroodi et al., 2020). The Arabian Gulf, in particular, harbors diverse communities of actinobacteria from sponge symbionts and sediment-associated microorganisms, some of which display thermophilic or thermotolerant characteristics (Lazar et al., 2022).

### 5.2 Coupled thermophily and halophily

A distinctive feature of thermophilic bacteria in the Arabian Gulf is the frequent coexpression of both thermophilic and halophilic traits, reflecting adaptation to the region’s dual extremes (Dutta et al., 2022). Halophilic thermophilic bacteria have evolved osmoadaptation strategies that allow survival in both high-temperature and high-salinity environments. These organisms utilize specific organic osmolytes, ion transport mechanisms, and protein stabilization pathways that collectively enable homeostasis under combined temperature and salinity stress. The biotechnological significance of these coupled extremophiles is substantial, as they offer biocatalytic opportunities unmatched by organisms adapted to single extreme stressors.

### 5.3 Thermophiles across the Gulf Cooperation Council (GCC) countries

#### 5.3.1 Thermophilic life in Saudi Arabia

Microorganisms known as thermophiles flourish in hot, humid settings like hydrothermal vents and hot springs. Thermophiles may be found at a number of hydrothermal vents and hot springs in Saudi Arabia (Al-Daghistani et al., 2021).

One such location is the Farah Garan East prospect, which is situated in Saudi Arabia’s Precambrian Arabian Shield in the southeast. Dolomite-talc breccia bodies in the shape of funnels are present in the prospect; these are thought to represent undersea hot-spring vent breccias, and they crop out close to the north and south ends of the dolomite strata. From these vents, aprons of exhalative dolomite thin laterally outward. East of and structurally under the hot spring vents, discordant and concordant zones of carbonate-altered metavolcanic rocks most likely indicate hydrothermal circulation paths over networks of fractures in volcanic rocks next to these Precambrian submerged hot spring vents (Damer and Deamer, 2020). The carbonate-rich rocks of the hot-spring assemblage include minerals in outcrops and geochemically anomalous quantities of gold, silver, copper, lead, zinc, arsenic, antimony, and tellurium (Mahmoud A. S. et al., 2021; Mahmoud H. et al., 2021). The biotechnologically significant enzymes of the thermophiles present in these settings have been studied. Studies have also been conducted on the geochemistry of Saudi Arabia’s hot springs (Alshehri et al., 2022).

Khalil (2011) pioneered the study of thermophiles in Saudi Arabia and has extensively investigated the integrated analysis of thermophilic microbial diversity from the geothermal environments of Al Khobar, Saudi Arabia, demonstrating his extensive contribution to the discovery, characterization, and genomic elucidation of high-value biotechnological resources. Three newly examined thermophilic strains (M4, M5, and Sh3) were shown to produce thermostable lipase, cellulase, and amylase, enzymes capable of degrading fats, cellulose, and starch under high temperature conditions, thereby underscoring their potential for industrial processes such as detergent formulation, textile treatment, and molecular biology applications that require robust catalytic performance.

Complementing his scientific research, he has also conducted detailed genomic investigations of *Anoxybacillus flavithermus* AK1, a bacterium thriving at 55–60 °C with a compact 2.63 Mb, high GC genome enriched in protein coding genes and containing 75 unique loci associated with heat resilience, pollutant degradation, and specialized metabolic pathways relevant to sugar processing, biofuel production, and novel compound biosynthesis (Khalil A. et al., 2018; Khalil et al., 2019).

The study further expands scientific understanding of *Brevibacillus borstelensis* AK1, whose approximately 4,000 protein coding genes and broad thermal growth range (45–70 °C) highlight its strong potential for bioremediation, particularly in the degradation of hydrocarbons and synthetic polymers such as plastics (Khalil A. B. et al., 2018).

Together, these findings not only illuminate the metabolic versatility and ecological significance of thermophiles from an underexplored region but also establish a foundational resource for future biotechnological innovation, reflecting the researcher’s substantial role in advancing microbial genomics, environmental biotechnology, and the applied use of extremophiles.

#### 5.3.2 Thermophilic life in Qatar

Halo-thermophilic bacteria and cyanobacteria adjacent to halophytic plants in the Sabkhas of Qatar was investigated and the study revealed the significant presence of thermo-halophilic bacteria, particularly near Limonium axillare, and the lowest bacterial populations near Suaeda virmiculata (Al-Thani and Yasseen, 2021).

The isolated strains were identified as Gram-positive rods, mainly *Bacillus thuringiensis* or *Bacillus cereus*, capable of forming endospores. Additionally, only cyanobacteria, particularly Anabaena and Nostoc, grew when Sabkhan soils were transferred to the laboratory under natural conditions, with some producing biofilms, heterocysts, and akinetes. The study suggested that these microorganisms may support halophyte growth by alleviating salt stress and other extreme environmental conditions in the saline Sabkhan soil (Joint FAO, 2018). The study explores the role of microbial communities in desert ecosystems, particularly in the Arabian Gulf region, including Qatar.

It discusses the ecophysiology of halophytes and their adaptations to different soils, highlighting differences in their habits and salt tolerance mechanisms. The research methodology, sampling, soil analysis, and microbiological analysis are detailed, emphasizing the importance of understanding and conserving microbial communities in desert and saline environments (Skariah et al., 2023). The study provides insights into microbial diversity and functionalities in the Sabkhas habitats in Qatar, enhancing soil microbiology, plant-microbe interactions, and ecosystem restoration. It highlights the importance of conserving desert ecosystems and the potential of extremophiles to mitigate harsh abiotic stresses (Mahmoud et al., 2023).

#### 5.3.3 Thermophilic life in United Arab Emirates

Numerous geothermal sites, such as hydrothermal vents and hot springs, are home to thermophiles, or species that flourish in hot temperatures. Although studies on thermophiles in the United Arab Emirates (UAE) are limited, studies on comparable geothermal systems in other areas have been carried out (Arbab et al., 2022; Rahman et al., 2022). Research has investigated the possibilities of hot springs around the globe, including those in Europe, the Caucasus, and the Azores, for biotechnological purposes. One such use is the enrichment of anaerobic heterotrophic thermophiles (Postec et al., 2023).

Furthermore, studies have looked into the geochemical circumstances for prebiotic chemistry in alkaline hydrothermal vents in shallow seas as well as the geomicrobiological specializations of hydrothermal systems in the Trans-Himalayan region. These studies have shed light on the diversity and distinctive features of thermophilic ecosystems (Mondal et al., 2022). These results show that thermophilic organisms may exist in geothermal environments and the importance of these ecosystems for biotechnological and geochemical research. Additional research on the geothermal sites in the United Arab Emirates may yield important insights on the thermophilic organisms in the area (Sundaram et al., 2023).

#### 5.3.4 Thermophilic life in Bahrain

Thermophiles are found to reside in a number of Bahraini hot springs. For their geomicrobiological specializations, the hot springs in the Indus and Nubra Valleys, located in eastern and northern Ladakh, respectively, have been investigated (Mishra et al., 2023).

Renowned for its unique ecological and mineralogical features, the Lotus Pond Spring is located in the eastern Ladakh region’s Puga Valley. Studies have also been conducted on the microbiome and ecology of a hot spring-microbialite system in the Trans-Himalayan Plateau (Roy et al., 2020). Using refractory substrates, the enrichment of anaerobic heterotrophic thermophiles from four Azorean hot springs revealed varying community composition and species abundances. Numerous minerals and trace elements that are found in hot springs are known to promote the formation of thermophiles. Because thermophiles in hydrothermal vents and hot springs yield helpful enzymes for a variety of industrial processes, studying these microbes has significant biotechnological significance (Benammar et al., 2020).

#### 5.3.5 Thermophilic life in Kuwait

Thermophiles are creatures that are found in hot springs and hydrothermal vents, which are high-temperature settings. Kuwait has hot springs that sustain thermophilic life, despite the country’s lack of hydrothermal vents (Al-Mailem et al., 2010; Delgado Romero, 2021).

The geothermal activity in the area is responsible for the country’s hot springs, which have water temperatures as high as 65 °C. One such hot spring is found at Al Hamma. Thermophiles, or organisms that have acclimated to extremely high temperatures, such as bacteria and archaea, find a home in these hot spring habitats (Alqahtani, 2021). In addition to demonstrating how resilient and adaptable life can be in a variety of settings, the availability of thermophiles in Kuwait’s hot springs may have biotechnological implications because these organisms have special enzymes and metabolic pathways that can be useful for a range of industrial processes (Bahamdain, 2020).

The microbial diversity of hydrocarbonoclastic bacteria in Kuwaiti desert soil and their potential for hydrocarbon removal at high temperatures are described. “Moderately thermophilic, hydrocarbonoclastic bacterial communities in Kuwaiti desert soil was observed to enhance activity via Ca^2+^ and dipicolinic acid amendment” (Kansour and Al-Mailem, 2023). Research discovered that Kuwaiti desert soil samples, both uncontaminated and contaminated by oil, contained 10–100 cells g^–1^ of hydrocarbonoclastic bacteria that could thrive at 50 °C. After wet soils were incubated for 6 months at 50 °C, the populations were increased to 103 cells g^–1^ by enrichment (Nejidat et al., 2023).

The majority of these organisms, which belonged to the genus *Bacillus*, were somewhat thermophilic and grew better at 40–50 °C than at 30 °C. Additionally, species from the genera *Geobacillus, Brevibacillus, Aneurinibacillus, Isoptericola, Nocardia, Amycolatopsis, Marinobacter*, and *Paenibacillus* were discovered (Bhattacharya and Gupta, 2022). The variety of microorganisms in the soil suggests that it has a strong chance of removing hydrocarbons at high temperatures. By adding Ca^2+^ to the cultures, all examined isolates’ growth and capacity to consume hydrocarbons were significantly increased (up to 2.5 M CaSO_4_) (Ossai et al., 2020). When 8% w/v dipicolinic acid was added, the improved impact was considerably stronger. These new discoveries are helpful in recommending biotechnologies for the cleanup of waste hydrocarbons at a somewhat high temperature (Truskewycz et al., 2019).

### 5.4 Thermophilic bacteria isolated from various sites in the GCC countries

Microorganisms known as thermophiles, which are acclimated to high temperatures, have been identified from a variety of habitats inside the Gulf Cooperation Council (GCC). Because of their unusual adaptations and their biotechnological uses, these extremophiles have drawn attention from scientists. They have adapted to the harsh circumstances of their environments, which include high temperatures. The table below highlights some of the thermophilic bacteria isolated from a number of sites in the GCC regions.

Representative thermophilic microorganisms reported from diverse extreme environments across the Gulf Cooperation Council (GCC) countries. The table summarizes bacterial taxa isolated from Saudi Arabia, the United Arab Emirates, Qatar, Oman, Kuwait, and Bahrain, together with their corresponding habitats, including geothermal hot springs, sabkhas, petroleum reservoirs, desert soils, and oil-contaminated environments. The isolates demonstrate substantial taxonomic diversity and adaptation to high-temperature ecosystems, highlighting the GCC region as an important reservoir of thermophiles with promising applications in industrial biotechnology, hydrocarbon bioremediation, and thermostable enzyme production. Data compiled from published studies. A group of thermophilic bacteria species documented within diverse locations in the GCC countries is presented in Table 2.

**TABLE 2:** Thermophilic bacteria isolated from various sites in the GCC countries.

GCC countries	Some isolated thermophiles	Location
Saudi Arabia	*Anoxybacillus flavithermus, Bacillus cereus, Bacillus licheniformis, Bacillus thermoamylovorans, Pseudomonas aeruginosa* and *Enterobacter* sp., *B. licheniformis, B. subtilis., Brevibacillus borstelensis* AK1	Al-Ain Alhara, a thermal hot spring located 50 km southeast of the city of Gazan, Saudi Arabia, Bani Malik and Al Khawbah., Al-Khawbah, Bani Malik, and Al-Bozah hot springs
United Arab Emirates (UAE)	*Bacillus* sp.	local United Arab Emirates hot water streams
Qatar	*Anabaena* and *Nostoc genera*	Sabkhas area
Oman	*Bacillus licheniformis* RW-3 isolated, *Alkaliphilus* sp., *Caminicella* sp., and *Desulfobacterium* sp, *Microcoleus chthonoplastes, Spirulina subsalsa, Johannesbaptistia pellucida, Chroococcidiopsis* sp., *Aphanocapsa* sp., *Chroococcus* sp., *Gloeocapsa* sp., *Schizothrix* sp. and *Leptolyngbya* sp.	Rustaq hot spring in Oman, Hammam, Bowshar, Nakhl and Rustaq, Sultanate of Oman
Kuwait	*Amycolatopsis*, *Chelativorans*, *Isoptericola*, *Nocardia*, *Aeribacillus*, *Aneurinibacillus*, *Brevibacillus*, *Geobacillus*, *Kocuria*, *Marinobacter* and *Paenibacillus*	pristine and oil-contaminated Kuwaiti desert soils
Bahrain	*Thermoanaerobacter* sp., *Thermoanaerobium*, *Fervidobacterium*, an isolate resembling *Dictyoglomus* sp.	petroleum reservoirs located in Bahrain

### 5.5 Molecular and physiological adaptation mechanisms

#### 5.5.1 Protein stabilization and heat shock protein systems

Thermophilic bacteria in the Arabian Gulf employ elaborate molecular strategies to maintain protein structure and function at elevated temperatures. Heat shock proteins (Hsp), including Hsp70, Hsp90, and their archaeal homologues in thermophilic species, play critical roles in preventing protein aggregation, facilitating proper folding, and enabling cellular survival during thermal stress (Matarredona et al., 2020). These proteins function as molecular chaperones, assisting in the refolding of thermally denatured proteins and preventing irreversible damage to essential cellular machinery.

Additionally, thermophilic organisms synthesize thermoprotectant molecules including trehalose, glycerol, and other compatible solutes that stabilize protein three-dimensional structures and maintain membrane fluidity. The synthesis and regulation of these protective molecules are controlled by genes that have undergone adaptive evolution, enabling fine-tuned responses to temperature fluctuations within the Arabian Gulf’s dynamic thermal environment.

#### 5.5.2 Membrane lipid composition and cellular architecture

The lipid composition of thermophilic bacterial membranes differs substantially from their mesophilic counterparts, reflecting evolutionary optimization for thermal stability. Thermophilic organisms typically increase the ratio of saturated to unsaturated fatty acids, facilitate cross-linking between lipid molecules, and incorporate specialized lipids that provide enhanced thermal stability (Ando et al., 2021). These compositional adaptations maintain appropriate membrane fluidity and prevent either excessive rigidity or pathological fluidization at elevated temperatures.

Biomarker studies examining lipid profiles in Arabian Gulf sediments have identified characteristic lipid signatures consistent with thermophilic bacterial activity, providing paleoenvironmental records of historical thermal regimes and microbial community composition (Finkel et al., 2023). These lipid-based approaches complement molecular phylogenetic methods in documenting the long-term presence and ecological success of thermophilic bacteria in the region.

#### 5.5.3 Genomic adaptation and gene content variation

Comparative genomic analyses of thermophilic bacteria reveal systematic differences in genome organization, gene content, and regulatory elements compared to mesophilic organisms (Sysoev et al., 2021). Thermophilic species often possess expanded gene families involved in stress response, energy metabolism, and molecular chaperone synthesis, reflecting genomic optimization for survival in extreme thermal environments. The presence of multiple copies of genes encoding heat-stable DNA-binding proteins and thermostable DNA repair enzymes further underscores the genomic remodeling required to sustain life at elevated temperatures.

## 6 Case studies and research highlights from gulf countries: Saudi Arabian hot spring systems

The Saudi Arabian hot springs, particularly the Al-Majardah, Al-Khubah, and Al-Ardah systems, have yielded remarkable discoveries regarding thermophilic enzyme production. Screening of 84 bacterial isolates from these springs identified 78 strains producing one or more hydrolytic enzymes (Alrumman et al., 2018; Alrumman et al., 2019). Notably, the isolation of *Bacillus aerius*, *B. licheniformis*, and *B. sonorensis* from these springs represents significant contributions to our understanding of thermophilic diversity in Arabian geothermal systems. These strains demonstrated simultaneous production of α-amylase, protease, and lipase activities, with enzyme yields substantially enhanced through cultural condition optimization.

The application of kitchen waste as substrate for enzyme production by Saudi Arabian thermophiles exemplifies the circular economy potential of extremophile biotechnology. Maximum lipase production (5.0% w/v waste concentration) and α-amylase/protease productivity (7.0% w/v waste concentration) achieved through waste utilization demonstrates economic viability for commercialization (Alrumman et al., 2018). This research provides a template for bioeconomy development in petroleum-dependent Gulf nations seeking to diversify industrial applications of thermophilic microorganisms.

The table below highlights some of the thermophilic bacterial species from Saudi Arabia and associated regions and their key applications (Tables 3, 4).

**TABLE 3:** Thermophilic bacterial species from Saudi Arabia and associated regions.

Thermophilic bacteria	Growth conditions		Obtained location	References
	Temperature (°C)	pH		
*Bacillus thermocopriae* (KKU-AS21), *Bacillus sonorensis* (KKU-KS2), *Brevibacillus borstelensis* (KKU-KS25) *Brevibacillus parabrevis* (KKU-MS8)	70–80	7.2–8.10	Hot springs, Jazan and Asir regions	(Alrumman et al., 2019)
*Streptomyces* sp. Al-Dhabi-1	37–55	6.0–11.0	Tharban hot spring in Asir region	(Al-Dhabi et al., 2016)
*Bacillus* spp., *Bacillus subtilis, Bacillus polymyxa, Bacillus licheniformis, Bacillus cereus, Bacillus mycoides Bacillus pumilus*	60	–	Soil from Al-Ahsa region	(Al-Humam, 2016)
*Actinobacteria isolates*	30–45	4.0–10.0	Desert environments, Saudi Arabia	(Shinwari et al., 2013)
*Bacillus licheniformis, Geobacillus stearothermophilus, Brevibacillus borstelensis* AK1, and *Anoxybacillus flavithermus* AK1	50	7.0	Hot spring, Saudi Arabia	(Abdulsalam et al., 2026)
*Streptomyces* sp. DA3–7	Up to 40	3.0–11.0	Desert of the Riyadh region	(Nithya et al., 2018)
*Bacillus subtilis, Bacillus licheniformis, Bacillus cereus*	37–45	5.0–9.0	Salty soil from Farasan island, Al-Harth Hot Spring, Salty water from Farasan Island, Jazan	(Al-Thobaiti et al., 2021)
*Nocardiopsis synnemataformans* NBRM9	25–60	5.0–13.0	Soil from the northern border region	(El-Sayed et al., 2024)
*Bacillus* sp., *Brevibacillus borstelensis*	55–60	7.5–8.5	Al-Khoba hot spring	(Khalil, 2011)
*Deinococcus geothermalis*	55–60	7.5–8.5	Al-Arida hot spring	(Khalil A. et al., 2018)
*Anoxybacillus flavithermus* AK1	55–60	–	Al-Ain Alhara hot spring of Gazan	(Khalil A. B. et al., 2018)
*Brevibacillus borstelensis* AK1	45–70	–	southeast of the city Gazan hot spring	(Khalil et al., 2019)

**TABLE 4:** Thermophilic bacterial species from Arabian Gulf and key applications.

Species	Optimal temperature (°C)	Optimal pH	Key application(s)	References
*Bacillus aerius, Bacillus licheniformis*	65	7.5–8.5	α-amylase protease production Lipase, protease production	(Alrumman et al., 2018)
*Parageobacillus caldoxylosilyticus, Anoxybacillus geothermalis, Geobacillus kaustophilus*	75 72 70	7.0	Long-chain alkane Oil degradation and bioremediation Cellulase, paraffin degradation	(Adlan et al., 2020)
*Thermococcus* sp.	85	7.0	Xylanase, cellulase production	(Gavrilov et al., 2016)
*Metallosphaera sedula*	75	2.0–4.5	DNA polymerase, biomining	(Ilyas et al., 2025)
*Geobacillus thermoglucosidasius*	60–65	7.0–8.0	Thermostable protease	(Suleiman et al., 2020)
*Bacillus* sp., *Brevibacillus borstelensis*	55–60	7.5–8.5	Lipase, cellulose	(Khalil, 2011)
*Deinococcus geothermalis*	55–60	7.5–8.5	α-amylase	(Khalil A. et al., 2018)
*Anoxybacillus flavithermus* AK1	55–60	–	Industrial processes and high-temperature bioremediation	(Khalil A. et al., 2018)
*Brevibacillus borstelensis* AK1	45–70	–	polyethylene (plastic) and long-chain hydrocarbons degradation	(Khalil A. B. et al., 2018)

Thermophilic bacteria isolated from Saudi Arabia’s geothermal and extreme environments exhibit remarkable physiological diversity and biotechnological potential. The table summarizes representative species, their optimal growth temperatures and pH ranges, isolation sites, and references. Isolates belonging to *Bacillus*, *Geobacillus*, *Anoxybacillus*, *Brevibacillus*, *Streptomyces*, *Nocardiopsis*, and *Deinococcus* were recovered from hot springs, desert soils, saline habitats, and geothermal regions. Their adaptability to harsh conditions underscores Saudi Arabia’s rich thermophilic biodiversity and highlights their promising applications in industrial biotechnology, enzyme production, and environmental bioremediation.

Representative thermophilic bacteria isolated from the Arabian Gulf exhibit diverse physiological characteristics and significant biotechnological potential. The table summarizes their optimal growth temperatures, pH ranges, and key applications, including the production of thermostable enzymes such as α-amylase, protease, lipase, cellulase, xylanase, and DNA polymerase, alongside hydrocarbon degradation, plastic biodegradation, biomining, and bioremediation. These findings demonstrate the industrial and environmental value of Gulf thermophiles, emphasizing their importance as sustainable biological resources for biotechnology and high-temperature industrial applications.

## 7 Evolutionary origins and biogeography of thermophily

### 7.1 Early emergence of thermophilic lineages

The evolutionary history of thermophily has been proposed to extend to the earliest branches of bacterial phylogeny, with evidence suggesting that thermophily may have characterized the last universal common ancestor or early primitive bacteria (Leng et al., 2023). Deep-branching thermophilic bacteria isolated from deep-sea hydrothermal vents display phylogenetic relationships consistent with ancient divergence, occupying basal positions within major bacterial lineages. These findings imply that thermophilic adaptation represents an ancestral microbial strategy that has been repeatedly lost during colonization of cooler environments.

### 7.2 Hydrothermal vent thermophiles and marine biogeography

Hydrothermal-vent studies from the Gulf of California and the Mediterranean provide comparative evidence that temperature and geochemistry can structure microbial niches (Thakur et al., 2026). However, differences in hydrography, geochemical composition, connectivity, and sampling depth prevent direct extrapolation to the Arabian Gulf (Rakib et al., 2021). These studies are therefore used only to formulate testable hypotheses. Rizzo et al. (2022) highlight that evidence for comparable vent-associated biogeographic patterns in the Arabian Gulf is currently insufficient, and targeted sampling of thermally influenced sediments, subsurface reservoirs, and geothermal sites is required.

### 7.3 Temperature-driven community assembly in stratified marine environments

Recent investigations of microbial communities in hydrothermally active marine sediments demonstrate that temperature-energy interactions fundamentally structure microbial assemblages, with archaea dominating communities at temperatures exceeding 45 °C while bacteria predominate in cooler zones. These temperature-driven compositional shifts occur independently of geographic location or specific habitat characteristics, suggesting universal thermodynamic constraints on microbial community assembly (Lagostina et al., 2021). Arabian Gulf thermophilic communities likely follow similar organizational principles, with temperature-dependent transitions occurring between different bacterial and archaeal populations at corresponding thermal thresholds.

### 7.4 Coupled halophily and thermophily in Arabian Gulf microorganisms

#### 7.4.1 Evolutionary co-adaptation to multiple extreme stressors

Thermophilic bacteria and archaea in the Arabian Gulf frequently exhibit halophilic characteristics, reflecting evolutionary pressure to simultaneously tolerate the region’s characteristic high temperatures and elevated salinity (Dutta et al., 2022). These halophilic thermophiles have developed sophisticated osmoadaptation strategies, accumulating intracellular solutes (especially potassium chloride and organic osmolytes) that maintain osmotic balance while preserving macromolecular function. The genomic basis for this dual extremophily remains incompletely characterized but appears to involve coordinated upregulation of genes encoding ion transporters, osmolyte biosynthetic enzymes, and stress-responsive proteins.

#### 7.4.2 Salt-resistant thermostable enzymes

Enzymes derived from halophilic thermophilic bacteria of the Arabian Gulf regions display exceptional stability across wide ranges of salt concentration, temperature, and pH, positioning them as ideal candidates for industrial biocatalysis (Dutta et al., 2022). These “extremozymes” maintain catalytic activity in high-salt environments that denature conventional enzymes, enabling applications in oil recovery enhancement, biosurfactant production, and bioremediation of hypersaline oil field waters (Dutta et al., 2022).

#### 7.4.3 Exopolysaccharide production and marine biofilm formation

Halophilic thermophilic bacteria isolated from Arabian Gulf and Red Sea environments synthesize exopolysaccharides (EPSs) with unique structural and functional properties adapted to high-salinity, high-temperature marine conditions (Ibrahim et al., 2018). These biopolymers have demonstrated applications as biosurfactants, metal-binding compounds, and environmental bioflocculants, with production yields and properties optimized in extremophilic microorganisms (Sharma et al., 2026). The EPS-producing capacity of halophilic thermophiles, exemplified by *Halomonas malpeensis* strains isolated from coastal regions, represents an underutilized biotechnological resource for sustainable production of marine biopolymers.

Table 5 summarizes the reported production conditions for hydrolytic enzymes from Saudi Arabian hot-spring isolates.

**TABLE 5:** Reported production conditions for hydrolytic enzymes from Saudi Arabian hot-spring isolates.

Isolate	Enzyme	Maximum reported activity (U mL^–1^)	Reported optimum temperature (°C)	Initial pH	Incubation (hours)	Kitchen-waste concentration
*Bacillus aerius*	α-Amylase	81.43	55–60	7.5–8.5	72	7% (w/v)
*Bacillus licheniformis*	Protease	281.36	55–60	7.5–8.5	72	7% (w/v)
*Bacillus sonorensis*	Lipase	167.3	55–60	7.5–8.5	72	5% (w/v)

Reported production conditions and enzymatic activities of hydrolytic enzymes produced by thermophilic bacterial isolates from Saudi Arabian hot springs. The table summarizes the bacterial isolates, enzyme types, maximum enzyme activities, optimum production temperatures, initial pH, incubation periods, and kitchen-waste substrate concentrations required for enzyme production. All isolates exhibited optimal enzyme production at 55–60 °C and pH 7.5–8.5 after 72 hours of incubation, with protease from Bacillus licheniformis showing the highest activity (281.36 U mL^−1^). Data compiled from Alrumman et al. (2018).

## 8 Metabolic diversity and functional gene repertoires

### 8.1 Autotrophic and chemolithautotrophic metabolisms in thermophiles

Thermophilic bacteria in extreme marine environments often employ chemolithautotrophic metabolic pathways, oxidizing reduced inorganic compounds such as sulfide, elemental sulfur, hydrogen, and ammonia as primary energy sources (Lagostina et al., 2021). While direct documentation of these metabolic capabilities in Arabian Gulf thermophiles remains limited, thermophilic chemolithoautotrophs have been extensively characterized in Mediterranean hydrothermal vents and similar environments, where they dominate high-temperature zones (Rizzo et al., 2022). These metabolic capabilities enable thermophilic bacteria to colonize seemingly barren high-temperature niches, supporting complex microbial food webs and biogeochemical processes in thermally extreme environments.

### 8.2 Anaerobic pathways and redox-dependent thermophilism

Anaerobic thermophilic bacteria, including strict anaerobes and facultative anaerobes, have been isolated from geothermal and oil reservoir environments worldwide. These organisms utilize diverse electron acceptors including sulfate, iron, and various organic compounds, enabling energy generation under oxygen-limiting conditions (Lazar et al., 2022; Gupta et al., 2021). The presence of anaerobic thermophilic pathways may explain the sustained activity of thermophilic bacterial communities in anoxic Arabian Gulf sediments and subsurface oil reservoirs, where oxygen availability is severely limited.

### 8.3 Mixotrophic nutritional strategies

Thermophilic bacteria capable of simultaneously utilizing both organic and inorganic carbon sources (mixotrophy) combine advantages of heterotrophic flexibility with chemolithoautotrophic independence from complex organic substrates (Matthews et al., 2023). This metabolic versatility provides thermophilic organisms with exceptional ecological flexibility, enabling proliferation under diverse nutrient regimes and growth conditions. Arabian Gulf thermophiles likely employ mixotrophic strategies, shifting metabolic pathways in response to fluctuating nutrient availability and substrate concentrations in oil-contaminated environments.

## 9 Extremozymes and industrial biotechnological applications

### 9.1 Thermostable cellulases and lignocellulose degradation

Thermophilic bacteria isolated from hot springs and geothermal environments demonstrate capabilities for producing industrially relevant thermostable cellulases with optimal activity at 50–60 °C and retention of substantial enzymatic activity across temperature ranges of 40–70°C (Arya et al., 2025). *Geobacillus stearothermophilus* strains isolated from Tapovan hot spring in India and related thermophilic bacteria show exceptional lignocellulolytic potential, efficiently degrading agronomic residues including wheat bran and sugarcane molasses.

While direct studies of Arabian Gulf thermophilic cellulase producers remain limited, the region’s thermophilic bacterial communities likely harbor similar enzymatic capabilities. The potential for bioethanol production and agricultural waste valorization through thermophilic cellulase systems has been extensively validated in thermophilic isolates from comparable environments.

### 9.2 Thermostable proteases

Thermophilic *Geobacillus* species, particularly *Geobacillus thermoglucosidasius*, produce thermostable proteases with maximum activity at 60–65 °C and pH 7–8 under 1% NaCl concentrations (Suleiman et al., 2020). These proteases retain substantial activity under diverse cultural conditions including variable salinity levels, pH ranges, and incubation temperatures, making them suitable candidates for detergent formulations, leather processing, and other industrial applications.

### 9.3 Alkaliphilic-thermophilic enzyme systems

Thermophilic bacteria capable of functioning under alkaline conditions produce extremozymes with exceptional robustness compared to conventional enzyme preparations. *Bacillus haynesii* and related species isolated from hot springs produce enzymes with high thermal stability and alkaline pH optima, supporting industrial applications in detergent manufacturing and food processing (Marín-Sanhueza et al., 2022). These coupled thermostability and alkaline functionality represent particularly valuable traits for detergent enzymes that must withstand both elevated wash temperatures and strong alkaline detergent formulations.

## 10 Bioremediation and environmental remediation applications

### 10.1 *In-situ* bioremediation of oil-contaminated sediments

The use of thermophilic bacteria for bioremediation of petroleum-contaminated Arabian Gulf sediments represents a promising strategy for ecological restoration (Sui et al., 2021). Thermophilic bacterial consortia, enriched from contaminated sediments and inoculated into oil-saturated soils, demonstrate progressive removal of hydrocarbons under aerobic and anaerobic conditions, with degradation rates exceeding 50–65% within the first month of treatment (Ali et al., 2020). Extended treatment periods show continued hydrocarbon consumption at rates of 14–24% monthly, with the highest numbers of degradative organisms correlating chronologically with maximum oil removal.

### 10.2 Thermophilic bioaugmentation strategies

Bioaugmentation using thermophilic bacterial isolates from Arabian Gulf oil fields represents a targeted approach to enhance bioremediation efficiency in hypersaline, high-temperature environments (Sui et al., 2021). Strains such as Bacillus subtilis and Pseudomonas species isolated from oil-contaminated Dehloran oil fields demonstrated superior performance in degrading diesel and crude oil at elevated temperatures (up to 50 °C) and salinity levels (10%), conditions that inhibit non-thermophilic bioremediators (Hosseini et al., 2024). These extremophile-based bioaugmentation approaches show substantially higher remediation effectiveness compared to conventional mesophilic bioremediation strategies, particularly in Arabian Gulf oil fields characterized by high *in-situ* temperatures and salinity.

### 10.3 Coupled bioremediation and biosurfactant production

Thermophilic bacteria and halophilic thermophilic organisms in the Arabian Gulf and adjacent regions produce biosurfactants that enhance hydrocarbon bioavailability and microbial access to recalcitrant compounds (Nikolova and Gutierrez, 2021). These microbial surfactants represent green alternatives to synthetic surfactants, offering improved environmental compatibility and biodegradability while maintaining superior performance in hypersaline, high-temperature petroleum remediation applications.

### 10.4 Oil and hydrocarbon degradation capabilities

#### 10.4.1 Thermophilic hydrocarbon degradation pathways

Thermophilic bacteria in the Arabian Gulf region display remarkable capability for degrading complex hydrocarbons at temperatures between 50 and 70 °C, significantly exceeding the degradation efficiency of mesophilic organisms (Adlan et al., 2020). Strains including *Geobacillus* and related genera produce alkane monooxygenase, alcohol dehydrogenase, lipase, and esterase enzymes that catalyze the stepwise oxidation of long-chain hydrocarbons into metabolizable intermediates. The efficiency of these degradation pathways is particularly striking for paraffin wax and waxy alkane fractions in crude oil, which are recalcitrant to biodegradation at lower temperatures.

*Geobacillus kaustophilus* and related strains isolated from Arabian Gulf seawater and contaminated sediments have demonstrated capacity to degrade up to 70% of paraffin wax compounds within 3 days of incubation at 70 °C, achieving complete degradation of high-molecular-weight alkanes (C37–C40) through production of specialized degradative enzymes (Adlan et al., 2020). These thermophilic degraders become progressively enriched in oil-contaminated reservoirs and sediments, where elevated in-situ temperatures create thermal niches favorable for their proliferation.

Additionally, research isolated a strain known as VP3, an aerobic, thermophilic, and halotolerant Gram-positive, spore-forming bacterium isolated from a geothermal oil field in Sfax, Tunisia. It grows optimally at 55 °C within a range of 37–65 °C and tolerates salinity up to 80 g L^−1^ NaCl, indicating strong adaptation to extreme environments. The bacterium demonstrates high metabolic versatility, completely degrading vanillic acid within 9 hours under optimal conditions. It can also degrade a wide range of aromatic compounds, including phenol and m-cresol. Additionally, VP3 utilizes crude oil and diesel as energy sources, highlighting its potential in hydrocarbon bioremediation and environmental biotechnology (Saidan et al., 2024).

Table 6 summarizes the hydrocarbon degradation by bacterial isolates from contaminated sediments in the Arabian Gulf.

**TABLE 6:** Hydrocarbon degradation by bacterial isolates from contaminated Arabian Gulf sediment.

Hydrocarbon	Isolate	Temperature (°C)	Initial concentration (ppm)	Reported degradation (%)	Incubation (days)	Sample origin
Naphthalene	*Rhodococcus quinshengi* TA13008	37	100	100	18	Contaminated Arabian Gulf sediment
Naphthalene	*Proteus mirabilis* T2A12001	37	100	> 9	18	Contaminated Arabian Gulf sediment
Naphthalene	*Brevibacillus brevis* T2C2008	37	100	87	18	Contaminated Arabian Gulf sediment
Pyrene	*Rhodococcus quinshengi* TA13008	37	100	56	18	Contaminated Arabian Gulf sediment
Pyrene	*Proteus mirabilis* T2A12001	37	100	Approximately 44	18	Contaminated Arabian Gulf sediment
Pyrene	*Brevibacillus brevis* T2C2008	25–37	100	< 36	18	Contaminated Arabian Gulf sediment

Thermophilic bacterial isolates from contaminated Arabian Gulf sediments demonstrated significant potential for hydrocarbon biodegradation under laboratory conditions. The table compares the degradation efficiencies of naphthalene and pyrene by *Rhodococcus qingshengii*, *Proteus mirabilis*, and *Brevibacillus brevis* under varying incubation conditions. Among the isolates, *Rhodococcus qingshengii* TA13008 showed the highest performance, achieving 100% degradation of naphthalene and 56% degradation of pyrene within 18 days. These findings highlight the promising application of thermophilic bacteria for the bioremediation of petroleum-contaminated marine environments (Al-Thukair et al., 2020).

#### 10.4.2 Multi-substrate degradation and metabolic versatility

Beyond alkane oxidation, thermophilic bacteria in the Arabian Gulf region demonstrate metabolic versatility encompassing degradation of naphthalene, pyrene, and other polycyclic aromatic hydrocarbons (Al-Thukair et al., 2020). Mixed bacterial consortia combining thermophilic species with distinct complementary metabolic capabilities show enhanced degradation of complex hydrocarbon mixtures, with degradation efficiency approaching 90–100% for single aromatic compounds and 60–80% for high-molecular-weight hydrocarbons under thermophilic conditions (Al-Marri et al., 2023).

This enzymatic versatility reflects decades of evolutionary adaptation to petroleum-rich environments within the Arabian Gulf region, particularly in association with major oil and gas production activities (Ali et al., 2020). Natural selection within oil-contaminated sediments has progressively enriched thermophilic bacterial populations with multiple degradative capabilities, creating effective bioremediation agents (Ali et al., 2020).

#### 10.4.3 Salinity-dependent degradation and halophilic synergism

The simultaneous operation of thermophily and halophily in Arabian Gulf bacteria has produced hydrocarbon-degrading organisms capable of performing effective bioremediation under conditions of high salinity and elevated temperature (Ghorbannezhad et al., 2022). *Bacillus subtilis* and related thermotolerant strains isolated from hypersaline oil fields demonstrate hydrocarbon degradation efficiency of 55.5–77.2% for high-molecular-weight compounds (pyrene and tetracosane) at 5% NaCl and 50 °C, substantially exceeding performance of non-halophilic thermophiles.

## 11 Pharmaceutical and biotechnology applications

The polymerase chain reaction (PCR) technique, essential in molecular biology, utilizes Taq polymerase from the thermophilic bacterium *Thermus aquaticus* (Alabhai et al., 2025). This single discovery has revolutionized molecular diagnostics and biotechnology research, demonstrating the transformative potential of thermophilic enzymes in medical diagnostics and research applications.

Immobilized nucleoside 2-deoxyribosyltransferases (NDT) from thermophilic and psychrophilic bacteria have demonstrated remarkable potential for pharmaceutical applications. NDT enzymes from *Chroococcidiopsis thermalis*, when immobilized on chitosan beads, showed improved stability and recyclability, with a tenfold improvement in reaction yield over 20 consecutive cycles, enabling production of pharmaceutically relevant nucleosides like 2-fluoro-3-deoxyadenosine.

**FIGURE 2:**
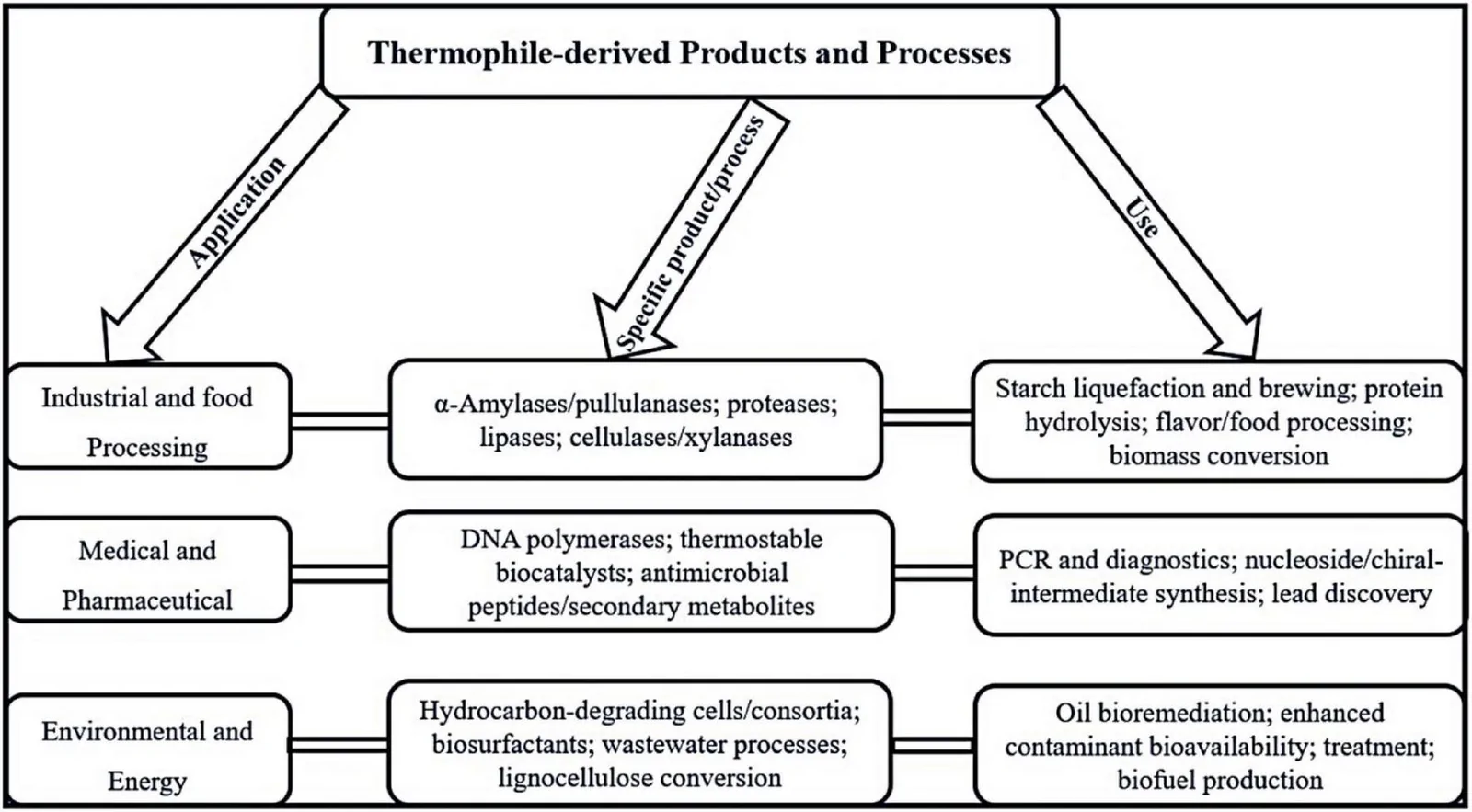
Thermophile-derived products and processes. Major applications of thermophilic microorganisms and their thermostable products. Examples are grouped by the product or process responsible for the application; enzyme-mediated uses are distinguished from whole-cell bioremediation and antimicrobial-compound discovery.

The utilization of thermophilic microbes in biotechnology is outlined in Figure 2.

## 12 Future perspectives and research priorities

### 12.1 Cultivation challenges and genomic characterization

The vast majority of thermophilic microorganisms from the Arabian Gulf remain uncultured, representing a substantial “microbial dark matter” of underexplored genomic and metabolic diversity (Schultz et al., 2023). Culture-independent methods including metagenomics, single-cell genomics, and genome-resolved metatranscriptomics have revolutionized our ability to characterize uncultured microbial populations, providing direct insight into functional capabilities without requiring laboratory cultivation (Sysoev et al., 2021). Applying these approaches systematically to Arabian Gulf thermophilic communities will substantially expand our knowledge of this biotechnologically important microbial reservoir.

### 12.2 Climate change impacts and thermal regime shifting

Projected increases in Arabian Gulf surface water temperatures as a consequence of global climate change will fundamentally alter the thermal ecology of the region, likely promoting further expansion of thermophilic bacterial communities and corresponding contraction of mesophilic populations (Lagostina et al., 2021). Understanding how thermophilic microbial communities will respond to ongoing temperature increases remains a critical research priority, with implications for marine ecosystem stability, fisheries productivity, and economic sectors dependent on the Gulf’s ecological services (López et al., 2024).

### 12.3 Biotechnological development and extremozyme engineering

The biotechnological potential of Arabian Gulf thermophiles remains substantially underdeveloped, with opportunities for expanded enzyme discovery, protein engineering, and biocatalytic applications (Abdul Rehman et al., 2026). Protein engineering approaches including directed evolution, rational design, and synthetic biology methods can further enhance the properties of thermophilic enzymes, enabling applications currently constrained by enzyme stability or catalytic efficiency limitations. Investment in comprehensive genomic screening, high-throughput enzyme characterization, and rational enzyme design targeting Arabian Gulf thermophiles could yield commercially significant biotechnological innovations.

### 12.4 Metagenomics-based ecosystem studies and systems biology

Recent advances demonstrate that omics-based research in the Arabian Gulf is steadily expanding, providing valuable insights into the region’s unique microbial ecosystems. Mahmoud and Jose (2017) characterized coral-associated viral communities, and Habibi et al. (2023) investigated the taxonomic composition, metabolic potential, and antimicrobial resistance profiles of coastal sediment microbiota. Fakhraldeen et al. (2023) examined the spatial distribution of bacterial and archaeal communities across Kuwaiti marine environments. More recently, microbial community structure has been correlated with environmental gradients within protected marine ecosystems, while comparative genomic analyses of *Anoxybacillus flavithermus* isolated from a Saudi Arabian hot spring have revealed strain-specific genetic features and biosynthetic potential (Khalil et al., 2019). Collectively, these investigations establish an important foundation for advanced microbial research in the region.

Despite these achievements, several critical knowledge gaps remain. Comprehensive, genome-resolved investigations focusing specifically on thermophilic microorganisms are scarce, geographical coverage across Gulf Cooperation Council countries remains fragmented, and only a limited number of predicted heat-stable biocatalysts have undergone experimental validation (Al-Awthan et al., 2024). Furthermore, large-scale surveys of Arabian Gulf sediments, water columns, geothermal habitats, and oil reservoir microbiomes are still lacking, restricting our understanding of thermophilic diversity, ecological functions, and contributions to regional biogeochemical cycling.

Thermophilic microorganisms produce robust enzymes with significant industrial value under extreme conditions. Because conventional cultivation captures only limited diversity, advanced culture-independent molecular techniques enable comprehensive microbial characterization, facilitating the discovery of novel thermophiles, thermostable enzymes, and functional pathways for biotechnology, environmental remediation, and sustainable industrial bioprocesses (Shomali and Danish-Daniel, 2024).

### 12.5 Bioinformatics mining, synergistic applications, and therapeutic potential of thermophiles in the Arabian Gulf: research priorities for the Arabian Gulf

Genome mining and machine-learning-assisted annotation can prioritize candidate extremozymes from cultured genomes and metagenome-assembled genomes, but predicted functions require confirmation by heterologous expression and biochemical assays (Sysoev et al., 2021; Shomali and Danish-Daniel, 2024). Comparative genomics of *A. flavithermus* AK1 illustrates the value of strain-resolved analysis, while regional shotgun-metagenomic datasets provide community resources that can be screened for genes associated with heat, salinity, and hydrocarbon stress (Khalil et al., 2019; Habibi et al., 2023; Fakhraldeen et al., 2023;; Fakhraldeen et al., 2025). Future work should therefore connect genome-resolved sampling to cultivation, transcript and protein measurements, and enzyme validation.

Applications that combine thermotolerance, halotolerance, biosurfactant production, and hydrocarbon degradation are particularly relevant to hypersaline petroleum-impacted environments. Their performance should nevertheless be tested under site-relevant mixtures of temperature, salinity, nutrients, and contaminants rather than inferred from single-stressor assays (Al-Thukair et al., 2020; Ghorbannezhad et al., 2022; Al-Marri et al., 2023). Similarly, antimicrobial peptides and other secondary metabolites from Gulf thermophiles should be described as promising leads, not established therapeutics. Purification, structural characterization, cytotoxicity, mechanism-of-action studies, and in vivo validation are needed before clinical or commercial claims are justified (Alrumman et al., 2019; Abdulsalam et al., 2026; Alhanbali et al., 2025).

## 13 Conclusion

Thermophilic bacteria represent an immense and partially untapped resource for biotechnological innovation, with applications spanning industrial enzymology, bioremediation, pharmaceutical production, and sustainable biofuel generation.

Thermophilic bacteria inhabiting the Arabian Gulf region represent an extraordinary biological resource with profound biotechnological potential yet to be fully realized. The combination of elevated temperatures, high salinity, hydrocarbon saturation, and geothermal features creates unique ecological conditions favoring evolution of specialized thermophilic communities possessing metabolic capabilities and enzyme properties unmatched by mesophilic counterparts. Key genera including *Bacillus*, *Geobacillus*, *Parageobacillus*, and *Anoxybacillus*, along with specialized archaeal thermophiles, demonstrate remarkable adaptation to Arabian Gulf conditions through sophisticated molecular, cellular, and metabolic strategies.

Current biotechnological applications of Arabian Gulf thermophiles focus primarily on industrial enzyme production, hydrocarbon bioremediation, and biosurfactant generation, with demonstrated commercial viability in multiple sectors. Case studies from Saudi Arabian hot springs and Gulf sediment environments illustrate concrete examples of successful enzyme discovery, characterization, and application. However, significant challenges remain regarding cultivation of unculturable thermophiles, optimization of enzyme performance under variable industrial conditions, and translation from laboratory discovery to commercial-scale implementation.

Future thermophilic bacteria research should prioritize comprehensive exploration of Arabian Gulf microbial diversity through advanced genomic and metagenomic approaches, development of synthetic biology tools for thermophilic strain engineering, and integration of thermophilic biotechnology into sustainable biorefinery concepts. Success in these endeavors requires collaborative effort among international research centers, Gulf-based institutions, and industrial partners, supported by appropriate regulatory frameworks and equitable benefit-sharing mechanisms. The thermophilic bacteria of the Arabian Gulf stand positioned to contribute significantly to sustainable development goals through renewable biofuel production, environmental remediation, and industrial biotechnology applications, while simultaneously advancing fundamental understanding of life’s ability to thrive at environmental extremes.

In conclusion, current knowledge of thermophilic bacteria from this region remains limited compared to global studies, suggesting substantial potential for novel discoveries. Practically, the integration of culture-independent approaches, including metagenomics, metatranscriptomics, and genome mining, with conventional cultivation and enzyme-screening workflows will allow researchers to connect uncultured thermophilic diversity with recoverable strains, functional genes, and industrially useful biocatalysts. This combined strategy can accelerate the discovery of salt-tolerant thermostable enzymes, hydrocarbon-degradation pathways, biosurfactants, and bioactive metabolites specifically adapted to Arabian Gulf conditions.
